# *Pseudomonas synxantha* volatile organic compounds: efficacy against *Cadophora luteo-olivacea* and *Botrytis cinerea* of kiwifruit

**DOI:** 10.3389/fpls.2024.1398014

**Published:** 2024-05-08

**Authors:** Alessandra Di Francesco, Farwa Jabeen, Núria Vall-llaura, Erica Moret, Marta Martini, Rosario Torres, Paolo Ermacora, Neus Teixidó

**Affiliations:** ^1^ Department of Agriculture, Food, Environmental and Animal Sciences, University of Udine, Udine, Italy; ^2^ Postharvest Programme, Institute of Agrifood Research and Technology (IRTA), Lleida, Spain

**Keywords:** biocontrol, biofumigation, defense genes, grey mold, skin pitting, volatile metabolites

## Abstract

Volatile organic compounds (VOCs) are responsible for the antagonistic activity exerted by different biological control agents (BCAs). In this study, VOCs produced by *Pseudomonas synxantha* strain 117-2b were tested against two kiwifruit fungal postharvest pathogens: *Cadophora luteo-olivacea* and *Botrytis cinerea*, through *in vitro* and *in vivo* assays. *In vitro* results demonstrated that *P. synxantha* 117-2b VOCs inhibit mycelial growth of *C. luteo-olivacea* and *B. cinerea* by 56% and 42.8% after 14 and 5 days of exposition, respectively. *In vivo* assay demonstrated significant inhibitory effects. VOCs used as a biofumigant treatment reduced skin-pitting symptoms disease severity by 28.5% and gray mold incidence by 66.6%, with respect to the untreated control. BCA volatiles were analyzed by solid-phase microextraction coupled with gas chromatography–mass spectrometry (SPME–GC/MS), and among the detected compounds, 1-butanol, 3-methyl and 1-nonene resulted as the most produced. Their efficacy as pure synthetic compounds was assayed against mycelial growth of fungal pathogens by different concentrations (0.34, 0.56, and 1.12 µL mL^−1^ headspace). The effect of the application of VOCs as a biofumigant was also investigated as the expression level of seven defense-related genes of kiwifruit at different exposition times. The results indicated an enhancement of the expression of almost all the genes starting from 3 h of treatment. These results described *P. synxantha VOCs* characteristics and their potential as a promising method to adopt for protecting kiwifruit from postharvest diseases caused by *C. luteo-olivacea* and *B. cinerea*.

## 1 Introduction

In recent years, new strategies have been considered for postharvest fruit management in line with the new European Plant Health Regulation (EU 2016/2031) and jointly with the European Green Deal policy that aim to accelerate the transition to more sustainable systems of food production, reduce by 50% the use of pesticides, and increase by 25% the land areas used for organic farming by 2030. Because of all these health concerns, biopesticides based on antagonistic microorganisms have been discovered and firstly tested under controlled conditions, and subsequently in commercial and semi-commercial settings (Droby et al., 2016; Droby and Wisniewski, 2018; Teixidó et al., 2022).

Numerous biological control agents (BCAs), particularly bacteria belonging to *Pseudomonas* spp. and *Bacillus* spp., are able to produce volatile metabolites known for their antifungal efficacy (Gotor-Vila et al., 2017; Toffano et al., 2017). These metabolites, commonly known as volatile organic compounds (VOCs), can act directly or indirectly against fungal pathogen development (Toral et al., 2021).

The strain *P. synxantha* 117-2b has already shown a great ability to produce secondary metabolites (Di Francesco et al., 2023) and, for this reason, has been studied and tested in the present work as an alternative method to control kiwifruit postharvest diseases. Usually, microbial VOCs are alcohols, alkanes, acids, or ketones, characterized by their low molecular weight (Calvo et al., 2020). Volatile metabolites are produced by microorganism biosynthetic pathways as integral components of secondary metabolism (Che et al., 2017). VOCs could be potentially used with success as gaseous fruit postharvest treatment in a process defined as “biofumigation” (Di Francesco et al., 2015).

Kiwifruit can be stored up to 5 months at 0°C and a relative humidity (RH) of 92%–95% at normal refrigeration (NR) or for an extended period in controlled atmosphere (CA) (Di Francesco et al., 2018). During the storage period, fungal infections are a major problem for kiwifruit, causing serious economic losses (Niu et al., 2023). *Botrytis cinerea* is the causal agent of kiwifruit gray mold (Li Z. X. et al., 2023), identified as one of the primary causes of fruit losses (almost up to 30%) mainly during the export phase (Hyun et al., 2022). Conversely, *Cadophora luteo-olivacea*, a re-emerging fungal pathogen associated with kiwifruit skin-pitting symptom (Di Francesco et al., 2023) or side rot of pears (Wenneker et al., 2016) and apples (Köhl et al., 2018), is considered less harmful because of its latent behavior and sporadic emergence. However, the pathogen can infect fruits during the developmental stage, remaining latent during harvest, and appearing after 3–4 months of cold storage causing important economic repercussions for fruit postharvest industry (Di Francesco et al., 2022).

Concerning all these considerations, the present study has the aim to (i) evaluate *Pseudomonas synxantha* strain 117-2b VOCs’ efficacy against kiwifruit postharvest fungal pathogens, *B. cinerea* and *C. luteo-olivacea*, by *in vitro* and *in vivo* assays; (ii) biochemically characterize bacteria VOCs by using headspace solid-phase microextraction coupled with gas chromatography–mass spectrometry (SPME–GC/MS) and verify the effect of single synthetic pure compounds on the mycelial growth of the target pathogens; and (iii) check the effect of fruit biofumigation with *P. synxantha* VOCs, combined with curing, on the expression of kiwifruit defense-related genes.

## 2 Materials and methods

### 2.1 Kiwifruit

Kiwifruits *cv* “Hayward” [*Actinidia deliciosa* (A. Chev.) C.F. Liang & A. R. Ferguson] were harvested at commercial maturity (7.2° Brix) from an orchard under integrated pest management (IPM) production system located in the Friuli Venezia Giulia (FVG) region (46°02′19.08″N, 12°57′33.66″E), Italy. Fruits were selected apparently healthy, homogeneous in size, and free of lesions and stored at 0°C for 5 days with 92% RH until use.

### 2.2 Microorganisms

*Botrytis cinerea* (Bc13), *C. luteo-olivacea* (Cad21), and *P. synxantha* (117-2b) belonged to the mycological collection of University of Udine—Di4A. *Cadophora luteo-olivacea* Cad21 was isolated from the tissue of symptomatic kiwifruit and molecularly characterized (Di Francesco et al., 2023). *Pseudomonas synxantha* 117-2b was originally isolated from the surface of kiwifruits and subsequently molecularly characterized (Di Lenarda et al., 2010). *Botrytis cinerea* Bc13 was isolated from infected kiwifruit, purified, and morphologically characterized. The fungal pathogens were cultivated on potato dextrose agar (PDA, 39 g L^−1^) (Oxoid, UK) at 25°C, 1 and 2 weeks before the experiments, for *B. cinerea* and *C. luteo-olivacea*, respectively. *Bacillus pumilus* QST2808, the active component of the commercial product Sonata^®^, was purchased from the NRRL (Northern Regional Research Laboratory, IL, USA) since its effectiveness against the target pathogens was tested in our previous study (Di Francesco et al., 2023) and it was used as a positive control in the present work. For the assays, both bacterial strains were grown on nutrient agar (NA, 13 g L^−1^) (Oxoid, UK) at 25°C and the cells from 2-day-old NA plates were suspended in potassium phosphate buffer (PPB, 70 mL of 0.2 M KH_2_PO_4_, 30 mL of 0.2 M K_2_HPO_4_, and 300 mL of deionized water v/v/v, pH 6.5) and adjusted to the final concentration of 1×10^8^ cells mL^−1^.

### 2.3 *In vitro* assay: bacterial VOCs’ efficacy

To verify *P. synxantha* 117-2b (Di Francesco et al., 2023) VOCs’ efficacy against *C. luteo-olivacea* (Cad21) and *B. cinerea* (Bc13) mycelial growth, a double Petri dish assay was performed as reported by Di Francesco et al. (2019). Plates were incubated at 25°C under dark conditions for 14 and 5 days for *C. luteo-olivacea* and *B. cinerea*, respectively. *Bacillus pumilus* QST2808 (1×10^8^ cells mL^−1^) was inoculated as reported above and used as a positive control. NA plates inoculated with 100 μL of sterile distilled water (SDW) represented the negative control. The sample unit of each treatment was represented by five replicates and the experiment was performed twice. The inhibition rate of mycelial growth was calculated using the equation (Dennis and Webster, 1971):


% inhibition = (d1 − d2)/(d1)∗100

where d1 and d2 are the control colony and the treated colony diameters, respectively.

### 2.4 Bacterial volatile compound analysis

To identify VOCs produced by *P. synxantha* (117-2b) (Di Francesco et al., 2023), sterile glass vials containing 10 mL of NA were inoculated with 50 μL of *P. synxantha* suspension (1×10^8^ cells mL^−1^). Vials were incubated at 25°C for 2 days and then the VOCs were extracted and analyzed by a gas chromatograph (7890B Agilent Technologies, Santa Clara, USA) coupled with a mass spectrometer detector (5977A, Agilent Technologies, Santa Clara, USA) equipped with an autosampler for SPME automatic injections (PAL RSI 85, CTC). An SPME fiber with 50/30 μm DVB/CAR/PDMS coating was selected and conditioned before use. The method used for the extraction and absorption of volatiles provided an incubation of the vial at 40°C for 5 min, followed by fiber exposure of 30 min at the same temperature. For the desorption of the volatiles, the fiber was introduced into the injector at 250°C for 5 min. The separation of analytes was performed on a 60-m-long fused silica capillary column, with an internal diameter of 0.50 mm and a polar stationary phase (DB-WAX, Agilent Technologies, Santa Clara, USA) with a film thickness of 0.5 µm. The GC oven temperature program was as follows: 40°C for 3 min, raised from 40°C to 220°C by a constant ramp of 4°C min^−1^; 220°C for 1 min, raised from 220°C to 250°C at 10°C min^−1^; and 250°C for 1 min. Helium was used as carrier gas with a constant column flow rate of 1.5 mL min^−1^. The transfer line temperature was maintained constant at 250°C. Ion source and quadrupole were set at 230°C and 150°C, respectively. Upon exiting the column, compounds were ionized via electron impact at 70 eV and detected with a quadrupole mass spectrometer in the range of a mass/charge ratio (*m*/*z*) from 35 to 500. Chromatograms were processed and analyzed by Mass Hunter (Agilent, USA). Compound identification was achieved by comparing the spectra with the NIST Standard Reference Database (NIST2014) and by linear retention indices (LRI) calculated under the same chromatographic conditions, injecting C7–C30 n‐alkane series (Supelco, Milan, Italy). VOC content was reported as absolute peak area (Aprea et al., 2012). The sample unit consisted of four replicates.

### 2.5 Effect of synthetic volatile compounds on *C. luteo-olivacea* and *B. cinerea* mycelial growth

Two synthetic compounds (1-butanol, 3-methyl and 1-nonene) (Merck, Germany) identified as the main VOCs produced by *P. synxantha* 117-2b were selected and singularly used. For each pure compound, different concentrations (15, 25, and 50 µL, corresponding to 0.34, 0.56, and 1.12 µL mL^−1^ headspace, respectively) were settled on sterile filter paper (Whatman^®^, 90 mm Ø), previously placed inside the Petri dishes (90 mm Ø) containing PDA inoculated with fungal plugs (6 mm Ø) of each pathogen. The dishes were immediately double-sealed with Parafilm^®^ and incubated at 25°C for 14 and 5 days, respectively, for *C. luteo-olivacea* and *B. cinerea*. The effect of each compound was detected by measuring the diameters of each fungal colony (Youssef and Hashim, 2019). The control was represented by plates containing un-wet sterile filter papers. Each VOC concentration was represented by three plates per pathogen. The experiment was performed twice.

### 2.6 *In vivo* assay: kiwifruit biofumigation

Biofumigation assay was conducted to verify the effect of volatile compounds produced by the BCAs in increasing the fruit resistance to gray mold and skin-pitting during the cold storage. *In vivo* assay was conducted by using sterile plastic boxes (29 × 18 × 10 cm, L × W × H) containing 150 mL of NA at the bottom. Six hundred microliters of *P. synxantha* 117-2b suspension (1×10^8^ cells mL^−1^) was spread on the medium. The boxes were immediately double-sealed with Parafilm^®^ and incubated at 25°C for 2 days. Kiwifruits were surface-sterilized for 1 min with sodium hypochlorite (0.1% v/v), rinsed with distilled water for 1 min, and air-dried at room temperature. Fruits were artificially wounded (2 × 2 × 2 mm) at the equatorial part with a sterile nail and inoculated with 20 μL of conidial suspension of *C. luteo-olivacea* (Cad21) (1×10^5^ conidia mL^−1^) or *B. cinerea* (Bc13) (1×10^5^ conidia mL^−1^). Once dried, fruits were placed on sterile grills inside the boxes to be separated from the medium. Boxes, incubated at 15°C and 85% RH, were exposed to bacterial VOCs for 96 h. After the biofumigation, treated fruits were removed from the boxes and stored at 0°C for 3 months and 1 month for *C. luteo-olivacea* and *B. cinerea*, respectively. As a positive control, *B. pumilus* QST2808 was used as a biofumigant treatment. Boxes containing just NA were considered the negative control. Each sample unit was represented by three boxes (containing eight kiwifruit) for each pathogen and treatment.

### 2.7 Gene expression analysis

#### 2.7.1 Kiwifruit sampling

Kiwifruit peel was sampled from unwounded fruits treated with *P. synxantha* 117-2b VOCs (as described in Section 2.6), and collected at 3, 6, 12, 24, 48, and 96 h of biofumigation treatment using a sterile peeler. Fruit peel samples were immediately frozen in liquid nitrogen, lyophilized by freeze-drying (FD-10 Freezing Dryer, Lab kits, H.K.) under vacuum (<20 Pa) at a temperature of −36°C and freeze-dried for 7 days, and stored at −80°C until use. Fruits not exposed to bacterial VOCs were considered as control. Three boxes containing eight fruits were used for each VOC exposition time.

#### 2.7.2 RNA extraction and cDNA synthesis

Total RNA was extracted from the collected fruit peel samples using the Spectrum™ Plant Total RNA Kit (Sigma Aldrich^®^, DE, USA), according to the manufacturer’s protocol. The total RNA concentration and RNA quality were determined using a NanoDrop™ 2000 spectrophotometer (Thermo Scientific, DE, USA). The integrity of RNA was further confirmed with an agarose gel stained with GelRed™ Nucleic Acid Gel Stain (Biotium, Hayward, CA, USA). The cDNA synthesis was performed using 1 μg of total RNA and the SuperScript™ IV cDNA synthesis kit (Invitrogen, Thermo Fisher Scientific).

#### 2.7.3 qPCR analysis

The transcription levels of seven selected fruit defense genes (**Supplementary Table S1**) were assessed by qPCR using the 7500 Real-Time PCR System (Applied Biosystems, Foster City, CA). The reaction mixture consisted of the KAPA SYBR^®^ Fast qPCR Master Mix (Kapa Biosystems, Inc., Wilmington, USA), 100 nM of each primer, and 9 ng of cDNA. Thermal cycling conditions were as follows: (i) 10 min at 95°C for initial denaturation, (ii) followed by 40 cycles of 15 s at 95°C, and (iii) annealing at 60°C for 1 min. A final amplification cycle at 95°C for 15 s, 60°C for 1 min, 95°C for 30 s, and 60°C for 15 s was applied to determine the melting curve. A non-template control (NTC) was included in each run using DNase-free water instead of cDNA. The sequence of each targeted gene was identified in the kiwifruit genome database (https://kiwifruitgenome.org/), and specific primers were designed *de novo* (**Supplementary Table S1**) using the NCBI primer blast tool (https://www.ncbi.nlm.nih.gov/tools/primer-blast/). Primer efficiency was determined by the serial dilution method, using a mix of all cDNA samples as a template. Genes encoding for *ACTIN* and *18S RNA* were checked as independent reference genes, and *ACTIN* was selected for its constant expression among sample conditions. Relative gene expression was expressed as mean normalized expression (MNE) according to Muller et al. (2002), using *ACTIN* as a reference gene. Results were obtained from the average of three biological replicates.

### 2.8 Statistical analysis

Data were statistically handled by one-way analysis of variance (ANOVA) using MiniTab 17. Mean statistical comparison of the effect of VOCs on the fungal colony diameter and on the incidence and disease severity was carried out by using Tukey’s HSD test (α = 0.05), as reported by Abo-Elyousr et al. (2020). For gene expression analysis, comparison of the means within the control and treated samples was performed using the Student’s *t*-test (α ≤ 0.05), and that between different time points for control and treated samples was carried out using Tukey’s HSD test (*α ≤ 0.05, ** α ≤ 0.01).

## 3 Results

### 3.1 *Pseudomonas synxantha* 117-2b VOCs’ *in vitro* efficacy against kiwifruit fungal pathogens

**Figure 1:**
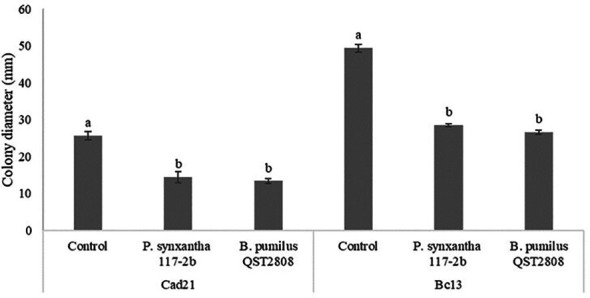
Effect of volatile compounds produced by *Pseudomonas synxantha* 117-2b and *Bacillus pumilus* QST2808 on the mycelial growth (mm) of *Cadophora luteo-olivacea* (Cad21) and *Botrytis cinerea* (Bc13). Fungal colony diameters (mm) were measured after 14 and 5 days at 25°C, respectively. Each value is the mean of five plates (replicates) ± standard error. Different letters represent significant differences among the treatments within each pathogen according to Tukey’s HSD test (α = 0.05).

The double Petri dish assay displayed the potential *in vitro* efficacy of VOCs produced by *P. synxantha* 117-2b against *C. luteo-olivacea* and *B. cinerea*. **Figure 1** reports the results of the experiment that showed a significant inhibitory effect of *P. synxantha* 117-2b and *B. pumilus* QST2808 VOCs against pathogens’ mycelial diameter. *Pseudomonas synxantha* 117-2b showed an inhibition of 56% and 42.8%, with respect to the relative control, against *C. luteo-olivacea* and *B. cinerea*, respectively. Likewise, *B. pumilus* QST2808 showed a significant inhibition, with respect to the relative control, against both fungal pathogens, of 52% and 44.9%, respectively.

### 3.2 SPME–GC/MS analysis of VOCs from *Pseudomonas synxantha* 117-2b

The volatile profile of *P. synxantha* 117-2b was investigated on NA medium through SPME–GC/MS (**Supplementary Table S2**). VOCs emitted by the bacterial antagonist exhibited a miscellaneous range of chemical compound classes, particularly alcohols (1-butanol, 3-methyl and phenylethyl alcohol), alkenes (1-nonene), and ketones (2-pentanone) (**Table 1**). VOCs that showed a higher absolute area (AA) were 1-nonene and 1-butanol, 3-methyl.

**Table 1:** Volatile organic compounds (VOCs) produced by Pseudomonas synxantha 117-2b at 25 °C after 48 h on Nutrient Agar (NA).

Compound	AA	RT
dimethyl sulfide	3.15E+06	5.51
**1-nonene**	3.54E+06	8.54
2-Pentanone	8.75E+04	9.57
Furan 2-propyl	3.39E+05	11.18
Methyl thiolacetate	1.85E+06	11.78
s-methyl propanethionate	3.06E+05	14.15
**1-butanol, 3-methyl**	1.13E+06	17.14
s-methyl 3-methylbutanethioate	1.87E+05	17.92
Dimethyl Sulfoxide	4.14E+04	29.81
Phenylethyl alcohol	7.38E+04	39.22

The values (AA, absolute area; RT, retention time) represent the average of the same compound analyzed on four vials more expressed and not detected on control vials.

### 3.3 Effect of synthetic volatile compounds on pathogens’ mycelial growth

**Figure 2:**
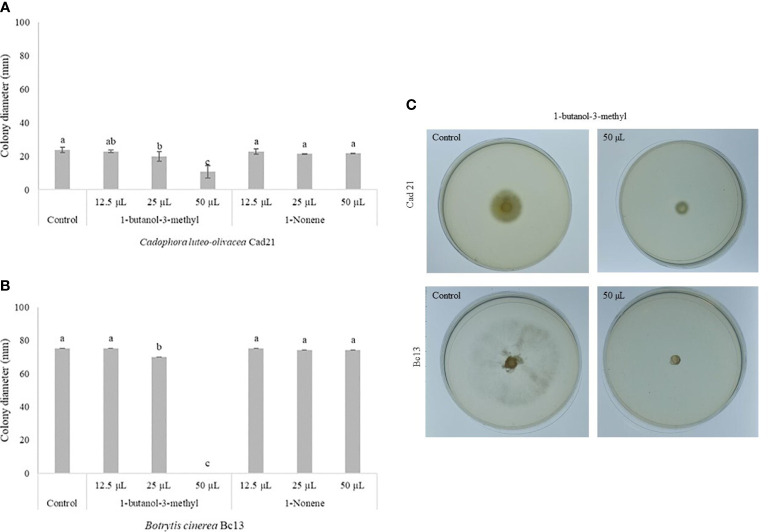
Effect of different concentrations (15, 25, and 50 µL, corresponding to 0.34, 0.56, and 1.12 µL mL^−1^ headspace, respectively) of synthetic volatile compounds mainly produced by *Pseudomonas synxantha* 117-2b on the mycelial growth (mm) of *Cadophora luteo-olicea* (Cad21) **(A, C)** and *Botrytis cinerea* (Bc13) **(B, C)**. Fungal colony diameters (mm) were measured after 14 and 5 days at 25°C, respectively. Each value is the mean of three plates (replicates) ± standard error. Different letters represent significant differences among the concentrations within each volatile compound according to Tukey’s HSD test (α = 0.05).

Among the detected VOCs, those with a higher AA such as 1-butanol, 3-methyl and 1-nonene were selected for assessing their effect on *C. luteo-olivacea* (**Figure 2A**) and *B. cinerea* (**Figure 2B**) colony growth. The compound 1-butanol, 3-methyl became more effective at 1.12 µL mL^−1^ headspace, showing an inhibition by 55.3% and 100% of *C. luteo-olivacea* and *B. cinerea* colony growth compared to the control, respectively (**Figure 2C**). The alkene 1-nonene did not exhibit any significant inhibitory effect against both fungal pathogens.

### 3.4 *In vivo* assay: efficacy of bacterial VOCs against kiwifruit postharvest molds

**Figure 3:**
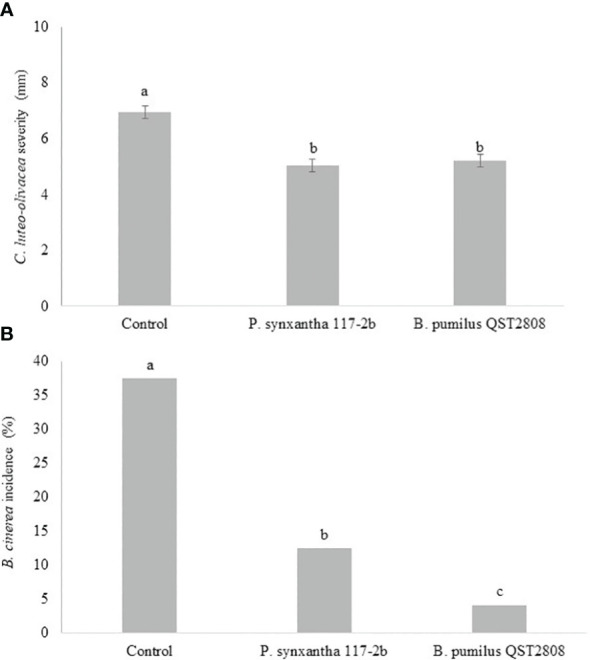
Effect of 96 h of exposition to VOCs produced by *Pseudomonas synxantha* 117-2b and *Bacillus pumilus* QST2808 on *Cadophora luteo-olivacea* severity (mm) **(A)** and *Botrytis cinerea* **(B)** disease incidence (%) on kiwifruit after 3 months and 1 month of cold storage (0°C), respectively. The symptoms caused by the two pathogens were differently detected for their different appearance on kiwifruit. The diameter of gray mold lesions was not detectable on kiwifruit, conversely for the skin-pitting. Moreover, *C. luteo-olivacea* incidence was not reported as not statistically different from the control.

VOCs produced by *P. synxantha* 117-2b showed a significant inhibitory effect against the targeted pathogens (**Supplementary Figures 1A, B**). Regarding *C. luteo-olivacea*, results were reported as severity (mm) of the disease (**Figure 3A**), and for *B. cinerea*, results were reported as percentage (%) of the disease incidence (**Figure 3B**), after 3 months and 1 month, respectively, of cold storage after 96 h of bacterial VOC biofumigation. The symptoms caused by the two pathogens were differently detected. The diameter of gray mold lesions was not easily detectable on kiwifruit, and conversely for the skin-pitting. Moreover, as regards *C. luteo-olivacea*, the incidence inhibition of the symptoms on fruits was not reported as not statistically different from the control. Regarding the skin-pitting symptoms, *P. synxantha* VOCs reduced the disease severity by 28.5% with respect to the control. For *B. cinerea*, the displayed inhibition of the disease incidence was 66.6% with respect to the control. VOCs produced by the reference strain *B. pumilus* QST2808, showed, in the case of skin-pitting, the same efficacy of the tested BCA, and conversely significantly higher in the case of gray mold incidence (90.3%).

### 3.5 Gene expression analysis of kiwifruit exposed to *P. synxantha* 117-2b VOCs

**Figure 4:**
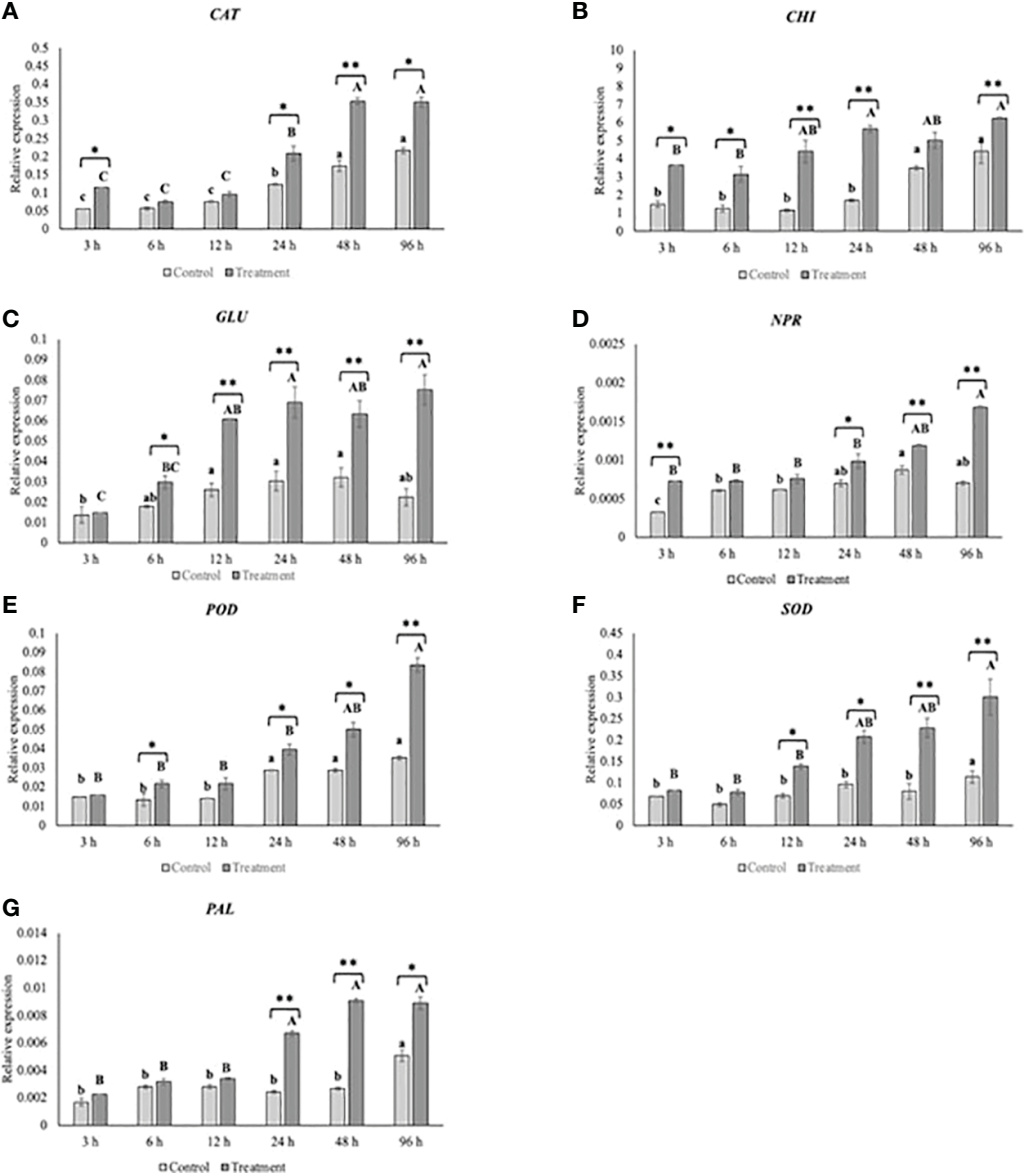
Effect of volatile organic compounds (VOCs) produced by *Pseudomonas synxantha* 117-2b on the expression level of *CAT* **(A)**, *CHI* **(B)**, *GLU* **(C)**, *NPR* **(D)**, *POD* **(E)**, *SOD* **(F)**, and *PAL* **(G)** genes in kiwifruit sampled after different time points (3, 6, 12, 24, 48, and 96 h) from bacterial VOC exposition. Data are expressed as the mean of three biological replicates ± standard error. For each time point (lowercase—control − uppercase—treatment), different letters indicate significant differences according to Student’s *t*-test (α ≤ 0.05). Asterisks denote significant differences between control and VOC-treated samples at the same time point (*α ≤ 0.05, ** α ≤ 0.01).

With the aim of determining the expression pattern of defense-related genes activated in kiwifruit as a response to *P. synxantha* 117-2b VOC exposition, the expression levels of several genes (**Supplementary Table S1**) at six different time points (3, 6, 12, 24, 48, and 96 h) after biofumigation were analyzed. Results revealed that the bacterial VOCs, in general, promoted the upregulation of the target genes (**Figure 4**). Specifically, the results for *CAT* expression levels (**Figure 4A**) revealed a significant increase in its expression at 3, 24, 48, and 96 h from the treatment with bacterial VOCs, compared to the control. Notably, the highest expression level was observed at 48 h post-treatment, which was 1.2-fold higher than the expression in the treated sample compared to the control. However, no significant differences in *CAT* gene expression compared to the control were observed after 6 and 12 h from the treatment. Moreover, a significant increase in the expression level of the *CAT* gene was also observed in the control samples from 24 to 96 h. Similarly, the expression of *CHI* gene (**Figure 4B**) started to increase significantly at 3 h from the treatment and continued to rise for 12 h. However, after the 12-h time point, the expression levels of the *CHI* gene were sustained, although a significantly higher expression occurred at 24 and 96 h, compared to the control. In the control fruit, the *CHI* gene showed a higher expression after 48 and 96 h compared to the earlier time points. In the case of *GLU* (**Figure 4C**), a significant increase in the expression was observed at 6 h from the treatment compared to the untreated fruit, increasing its expression for 96 h. No significant increase was detected in the control samples over time. The results of the *NPR* gene expression level (**Figure 4D**) revealed a significant increase along with the treatment time points and the maximum induction took place after 96 h from the treatment compared to the control. Conversely, no significant differences in *NPR* gene expression were observed at 6 and 12 h with respect to the control. The exposition of fruits to bacterial VOCs caused a significant increase in *POD* gene expression (**Figure 4E**) at 6 h, 24 h, 48 h, and 96 h compared to the control. Specifically, a notable upregulation occurred at 96 h, being 2.3-fold higher compared to the control. However, if compared to the control, no significant differences in *POD* expression at 3 and 12 h were detected. Regarding the *SOD* gene (**Figure 4F**), the treatment induced its expression levels after 12 h, and this pattern was sustained for 96 h. *SOD* gene expression increased in the control samples at 96 h compared to 0-h time point. The fruit *PAL* gene (**Figure 4G**) was notably upregulated after bacterial VOC exposition at 24, 48, and 96 h compared to the earlier time points. In contrast, no significant differences in *PAL* expression were observed in control samples, except for 96 h, in which gene expression levels were upregulated compared to the other time points.

## 4 Discussion

The present study aimed to find a sustainable solution to use during kiwifruit storage, for its preservation from skin-pitting and gray mold diseases by using a bacterial BCA instead of a synthetic fungicide. Many microorganisms including bacteria, yeasts, and fungi are VOC producers, and their biocontrol activity has been studied (Gotor-Vila et al., 2017; Di Francesco et al., 2020; Guo et al., 2023). Among bacteria, *Pseudomonas* has been studied as a potential BCA against a wide range of fungal pathogens, mainly for its ability to produce antifungal compounds (Sang and Kim, 2014; Aiello et al., 2019; Rojas-Solis et al., 2020; Di Francesco et al., 2023). Mukherjee et al. (2014) reported *P. synxantha* as a producer of bioactive compounds, including volatile compounds, effective against several microorganisms. The antagonistic activity through the production of secondary metabolites was already verified for *P. synxantha* strain 117-2b by Di Francesco et al. (2023). In the last few years, many studies on the different modes of action exerted by BCAs in the inhibition of pathogenic fungi have been conducted. Recently, the essential role of VOCs has been given more consideration (Toffano et al., 2017), specially for the potential use during the fruit postharvest phase as biofumigants. The present study has displayed the efficacy of the strain *P. synxantha* 117-2b against skin-pitting and gray mold of kiwifruit by producing VOCs, as it was already reported as an effective antagonist against *C. luteo-olivacea* through other modes of action (Di Francesco et al., 2023). In fact, using the double Petri dish assay, *P. synxantha* 117-2b showed a significant inhibition of the mycelial growth of the targeted pathogens *C. luteo-olivacea* (Cad21) and *B. cinerea* (Bc13) after the exposition to VOCs, reaching an inhibition of 56% and 42.8%, respectively. Its effectiveness was compared to *B. pumilus* QST2808, a strain selected for its high efficacy against *C. luteo-olivacea* in a previous study (Di Francesco et al., 2023). In *in vitro* assays, both treatments showed the same efficacy against the two target pathogens, making *P. synxantha* 117-2b a promising candidate for further investigation and potential application. The outcomes of our study are partially in agreement with the antifungal effect of VOCs produced by *P. synxantha* strain DLS65 applied against *Monilinia fructicola* and *Monilinia fructigena* in stone fruits (Aiello et al., 2019). In fact, Aiello et al. (2019) reported that the best performances of *P. synxantha* DLS65 were determined mainly by the mechanisms of action that involved the presence of bacterial living cells. In our case, VOCs produced by *P. synxantha* 117-2b also became effective in *in vivo* assays where an incidence reduction of almost 70% was registered for *B. cinerea.* In fact, the experiment combined two different strategies, curing and biofumigation, that can also contribute to an enhancement of kiwifruit disease resistance. Firstly, it was necessary to determine the *P. synxantha* 117-2b VOC profile by SPME–GC/MS analysis. Results revealed an array of volatile compounds belonging mainly to alcohols, aldehydes, and alkane classes.

Usually, the principal group of microorganism metabolites was formed by alcohols as reported in previous studies (Stoppacher et al., 2010; Rouissi et al., 2013). However, the applied methodology to collect and detect VOCs, as well as the growth medium, can strongly influence the results and often hinder the comparison between studies (Martins et al., 2010; Di Francesco et al., 2015). Comprehensively, *P. synxantha* VOC application as a biofumigant treatment during the curing phase slightly reduced *C. luteo-olivacea* symptoms without controlling the infection; thus, in this case, the treatment could be part of an integrated strategy to control skin-pitting. Instead, for *B. cinerea*, *P. synxantha* VOCs notably reduced the pathogen incidence (66.6%) compared to the untreated control. Perhaps, this effectiveness can be partly attributed to the overexpression of different fruit defense-related genes, such as *CAT*, *CHI*, *GLU*, *NPR*, *POD*, *SOD*, and *PAL*. In particular, the antioxidant role of genes such as *CAT*, *POD*, and *SOD* has been proven to be crucial for fruit disease resistance, also contributing to the response to biotic and abiotic stresses (Pan et al., 2020). Recent lines of evidence suggest that plants treated with resistance-inducing substances (e.g., chitosan) exhibit faster and stronger defense responses when infected by pathogens (Maya and Matsubara, 2013; Zhang et al., 2018). Our results showed that the bacterial VOCs’ exposition slightly increased the expression level of all targeted genes in treated kiwifruits. It is also known at the molecular level as bacterial VOCs could show up- and downregulation of gene expression (Wheatley, 2002).

However, the different genes showed different behavior patterns and a higher expression level at different time points. Kiwifruit *CHI*, *NPR*, *GLU*, and *CAT* genes showed an early response to bacterial VOC exposition starting their induction at 3 h from the treatment. Among the selected defense genes, the *CHI* gene represents one of the main pathogenesis-related (PR) members (Zhang et al., 2018) responsible for plant defense against pathogenic fungi by breaking down their cell walls (Li et al., 2013). Similar to the *CHI* gene, VOC exposition also triggered the expression of the *GLU* gene in kiwifruit. β-1,3-Glucanase plays a direct role in fungal defense by hydrolyzing fungal cell walls and promoting the formation of elicitors (Ebrahim et al., 2011). Our study reported that the increased expression of the kiwifruit *GLU* gene by VOC treatment was principally detected after 6 h compared to the control.

Regarding the *NPR* gene, it has been identified as a positive controller of defense response mediated by salicylic acid (SA) (Hong et al., 2016). In certain plants like *Arabidopsis* and grapes, the activation of *NPR* genes can occur within a day when exposed to SA or analogs, or to specific pathogens (Le Henanff et al., 2009). In our case, results showed a significant increase in *NPR1* expression levels after 3 h from treatment exposition, such as occurred for *CHI, GLU*, and *CAT* genes. In fact, a significant upregulation of key defense-related genes like *CAT, CHI, GLU, NPR, POD, SOD*, and *PAL* supports the hypothesis that *P. synxantha* 117-2b VOCs induced defense mechanisms in kiwifruit. However, the findings of our study demonstrated that the activation of each defense-related genes is strictly dependent on the different treatment times. Moreover, we also observed a gradual increase in the expression of *CAT, CHI, POD*, and *PAL* genes in the untreated samples over time. This phenomenon is likely due to the possible kiwifruit endogenous processes such as the natural senescence process that triggers the upregulation of defense-related genes, probably stimulated by the curing period (Wurms et al., 1997). Overall, our results demonstrated that VOCs produced by *P. synxantha* strain 117-2b can elicit a defense response in kiwifruit during the curing phase, supporting the obtained *in vivo* results, where the treatment reduced only gray mold incidence. Further studies are needed to better investigate the molecular mechanisms involved in the activation of defense response in kiwifruit and to implement the practical application of biocontrol agents and volatile metabolites in the postharvest phase to manage fruits through a sustainable strategy.

## 5 Conclusion

The findings of the present study provide new insights into the effects of VOCs produced by the strain *P. synxantha* 117-2b against *C. luteo-olivacea* and *B. cinerea*, both fungal postharvest pathogens of kiwifruit. The results showed how the production of VOCs plays an essential role in the antagonistic activity of a BCA and how their production can be strongly influenced by growth substrates. In addition, the biofumigant treatment of kiwifruits by *P. synxantha* 117-2b notably promoted the expression levels of fruit defense genes. For this reason, this BCA may be applied with the purpose of inhibiting the pathogen’s growth and, at the same time, increasing fruit defense responses. However, further investigation is necessary to understand the exact contribution of *P. synxantha* 117-2b in the control of kiwifruit postharvest pathogens and to improve its efficacy, which depended on the pathogen and the experimental conditions.

## Data availability statement

Data will be made available on request. Requests to access the datasets should be directed to alessandra.difrancesco@uniud.it.

## Author contributions

AD: Conceptualization, Data curation, Methodology, Writing – original draft, Writing – review & editing. FJ: Methodology, Writing – original draft, Writing – review & editing. NV-l: Data curation, Methodology, Supervision, Writing – review & editing. EM: Methodology, Writing – review & editing. MM: Supervision, Writing – review & editing. RT: Supervision, Writing – review & editing. PE: Project administration, Writing – review & editing. NT: Conceptualization, Methodology, Supervision, Writing – review & editing.
