# Supplementary material for: Pseudomonas synxantha volatile organic compounds: efficacy against Cadophora luteo-olivacea and Botrytis cinerea of kiwifruit
Source: Front Plant Sci. 2024 May 8;15:1398014. doi: 10.3389/fpls.2024.1398014 (PMC11109433; doi:10.3389/fpls.2024.1398014)

**Supplementary Figure 1.** Effect of 96 h of exposition to VOCs produced by *Pseudomonas synxantha* 117-2b on *Cadophora luteo-olivacea* severity (mm) (A) and *Botrytis cinerea* (B) disease incidence (%) on kiwifruit.


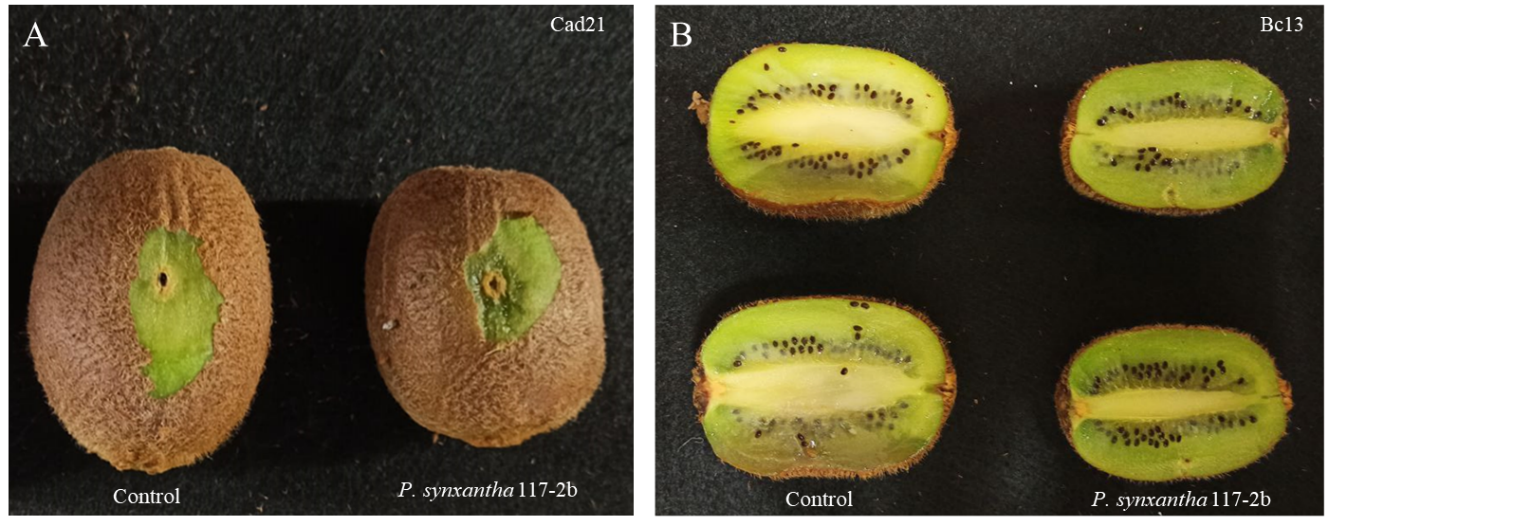

Supplement: Supplementary file 1 [file DataSheet_1.doc]
