# Supplementary material for: Pseudomonas synxantha volatile organic compounds: efficacy against Cadophora luteo-olivacea and Botrytis cinerea of kiwifruit
Source: Front Plant Sci. 2024 May 8;15:1398014. doi: 10.3389/fpls.2024.1398014 (PMC11109433; doi:10.3389/fpls.2024.1398014)
Supplement: Supplementary file 2 [file Table_1.docx]

| **Gene** | **Accession no.** | **Primer sequence 5´ --> 3´** | **Product size (bp)** | **Efficiency (%)** | | **Correlation** |
| --- | --- | --- | --- | --- | --- | --- |
| ***CAT*** | Ach21g051741.2 | F :ACAAGGCCGGTAAAGCACAT | 92 | 92.1 | | 0.974 |
|  |  | R : TCCGACCTTAACCGACTCCT |  |  |  |  |
| ***CHI*** | Ach08g341891.2 | F : AATCGCTGCTTTCTTCGCAC | 71 | 91.9 | | 0.992 |
|  |  | R : CGATTTCCGGGTTGTTGAGC |  |  |  |  |
| ***GLU*** | Ach24g103301.2 | F : ACCAGAACCTCTTTGACGCC | 145 | 95 | | 0.993 |
|  |  | R : TAAGTGGCTGCGTTGTCGAT |  |  |  |  |
| ***NPR*** | Ach01g326501.2 | F : GCCTTCTCCGATTCCAACGA | 110 | 97.3 | | 0.979 |
|  |  | R :CAGCGAAGGGATGCGATTTC |  |  |  |  |
| ***POD*** | Ach01g373131 | F : GGGGTGTGATGCTTCGGTTA | 97 | 94.3 | | 0.997 |
|  |  | R : GTCAAATCCATCTCCGGCCA |  |  |  |  |
| ***SOD*** | Ach03g419431.2 | F :ACTACAACAACGCCCTCCAG | 108 | 90.2 | | 0.983 |
|  |  | R : TGATATGACCTCCGCCGTTG |  |  |  |  |
| ***PAL*** | Ach08g066341.2 | F : ATCGTCGACCAAGTCCATCG | 143 | 102 | | 0.989 |
|  |  | R : TTGCTAGACGGGTGTTGTCC |  |  |  |  |
| ***18s RNA*** | AB253775 | F:GAATGACCCGCGAACTTGTC | 140 | 102 | | 0.997 |
|  |  | R:CGGGATTCGTTGTTTGACCG |  |  |  |  |
| ***ACTIN*** | EF063572.1 | F :CATCGTCCACAGGAAGTGCT | 193 | 94.9 | 0.977 | |
|  |  | R :GGCAAGGAGAGCCATCACAT |  |  |  |  |

**Table S1.** Primers used in qPCR analysis. Gene name, accession number, primer sequence, product size, efficiency and correlation values were specified. Target genes were identified from the kiwifruit genome database (https://kiwifruitgenome.org/). Both reference genes, *ACTIN* and *18s RNA* were identified from NCBI (<https://www.ncbi.nlm.nih.gov/genbank/>).
