# Supplementary material for: Pseudomonas synxantha volatile organic compounds: efficacy against Cadophora luteo-olivacea and Botrytis cinerea of kiwifruit
Source: Front Plant Sci. 2024 May 8;15:1398014. doi: 10.3389/fpls.2024.1398014 (PMC11109433; doi:10.3389/fpls.2024.1398014)
Supplement: Supplementary file 3 [file Table_2.doc]

| **Table S2.** All volatile compounds produced by NA (nutrient agar) and *Pseudomonas synxantha* 117-2b after 48 h of incubation at 25°C. The values represent the AA (absolute area), average of the same compound on four vials. | | | | |
| --- | --- | --- | --- | --- |
| **Compound** | | **RT** | **NA** | **117-2b / NA** |
| *dimethyl sulfide* | | 5.51 | 0.00 | 3.157.060,68 |
| *furan* | | 5.97 | 200.7537,42 | 118.328,48 |
| *Propanal, 2-methyl* | 6.17 | | 107.8628,24 | 0,00 |
| *acetone* | 6.23 | | 412.4761,92 | 484.175,83 |
| *tetrahydrofuran* | 6.95 | | 41.284.66 | 38.444,13 |
| *furan, 2-methyl* | 7.08 | | 79.477,68 | 51.214,88 |
| *butanal* | 7.19 | | 33.663,22 | 0,00 |
| *2-butanone* | 7.67 | | 209.402,70 | 164.977,35 |
| *Butanal, 2-methyl* | 7.94 | | 409.509,72 | 0,00 |
| *Butanal, 3-methyl* | 8.03 | | 531.682,46 | 0,00 |
| *Ethanol* | 8.39 | | 40.218,90 | 55.232,99 |
| *1-nonene* | 8.54 | | 0.00 | 3.546.204,79 |
| *Furan, 2-ethyl* | 8.92 | | 49.839,88 | 285.439,08 |
| *2-Pentanone* | 9.57 | | 0.00 | 87.476,45 |
| *2,3-Butanedione* | 9.59 | | 57.100,56 | 0,00 |
| *Furan 2-propyl* | 11.18 | | 0.00 | 339.322,82 |
| *Toluene* | 11.49 | | 339.428,63 | 182.288,92 |
| *Methyl thioloacetate* | 11.78 | | 0.00 | 1.845.902,99 |
| *Disulfide, dimethyl* | 12.54 | | 383.533,90 | 5.186.331,77 |
| *Hexanal* | 12.80 | | 23.853,07 | 0,00 |
| *s-methyl propanethionate* | 14.15 | | 0.00 | 306.998,19 |
| *1-Butanol* | 14.96 | | 48.342,51 | 0,00 |
| *Dodecane* | 16.68 | | 128.289,07 | 0,00 |
| *1-butanol, 3-methyl* | 17.14 | | 0.00 | 1.127.206,55 |
| *s-methyl 3-methylbutanethioate* | 17.92 | | 0.00 | 187.352,03 |
| *Furan, 2-pentyl* | 18.05 | | 25.255,99 | 77.364,62 |
| *Furan, 2-(methoxymethyl)* | 18.40 | | 9.556,84 | 22.333,28 |
| *Styrene* | 19.13 | | 29.373,28 | 38.523,43 |
| *Pyrazine, methyl* | 19.49 | | 17.975,52 | 23.114,63 |
| *Pyrazine, 2,5-dimethyl* | 21.45 | | 110.409,45 | 53.579,62 |
| *Pyrazine, trimethyl* | 24.22 | | 36.152,75 | 41.190,64 |
| *Acetic acid* | 25.77 | | 60.282,32 | 119.946,85 |
| *furfural* | 26.27 | | 203.851,81 | 13.562,93 |
| *1-hexanol, 2-ethyl* | 26.75 | | 31.529,74 | 55.546,45 |
| *Benzaldehyde* | 28.30 | | 3.404.131,82 | 41.578,64 |
| *Dimethyl Sulfoxide* | 29.81 | | 0.00 | 41.452,11 |
| *Ethanol, 2-(2-ethoxyethoxy)* | 30.93 | | 16.046,23 | 20.291,28 |
| *Butyrolactone* | 31.66 | | 14.854,28 | 28.142,64 |
| *2-acetylthiazole* | 32.02 | | 82.090,56 | 73.279,34 |
| *2-furanmethanol* | 32.13 | | 36.030,40 | 46.931,66 |
| *Acetophenone* | 32.19 | | 44.667,05 | 73.137,21 |
| *Benzyl alcohol* | 38.28 | | 27.463,63 | 15.334,15 |
| *Phenylethyl alcohol* | 39.22 | | 0.00 | 73.849,88 |
| *Benzyl nitrile* | 39.82 | | 53.015,20 | 43.499,53 |
| *Phenol* | 41.58 | | 16.324,34 | 14.470,39 |
| *Isophthalaldehyde* | 45.03 | | 100.333,28 | 30.624,49 |
| *Benzoic acid* | 51.32 | | 674.956,96 | 101.886,18 |
